# Effect of Functionalization of Graphene Nanoplatelets on the Mechanical and Thermal Properties of Silicone Rubber Composites

**DOI:** 10.3390/ma9020092

**Published:** 2016-02-02

**Authors:** Guangwu Zhang, Fuzhong Wang, Jing Dai, Zhixiong Huang

**Affiliations:** Key Laboratory of Advanced Technology for Specially Functional Materials of Ministry of Education, School of Materials Science and Engineering, Wuhan University of Technology, Wuhan 430070, China; daijing@whut.edu.cn (J.D.); zhixiongh@whut.edu.cn (Z.H.)

**Keywords:** silicone rubber, graphene nanoplatelets, functionalization, mechanical properties, thermal properties

## Abstract

This study investigated the effect of silane and surfactant treatments of graphene nanoplatelets (GnPs) on the mechanical and thermal properties of silicone rubber (SR) composites. GnPs were modified with aminopropyltriethoxysilane (APTES), vinyltrimethoxysilane (VTMS), and Triton X-100, and then the pristine GnPs and functionalized GnPs were individually incorporated into the SR. Compared with the pristine GnP/SR composite, the composites reinforced with modified GnP showed better tensile strength, elongation at break, and thermal conductivity properties due to better dispersion of modified GnPs and stronger interfacial interactions between the modified GnPs and matrix. The mechanical properties and thermal conductivity of the VTMS-GnP/SR composite were comparable to the properties of the Triton-GnP counterpart, but better than that of the APTES-GnP/SR composite. In addition, the VTMS-GnP/SR composite demonstrated the highest thermal stability and crystallization temperature among the four types of composites. The remarkable improvement of mechanical and thermal properties of the VTMS-GnP/SR composite was mainly due to the covalent linkage of VTMS-GnP with SR. The VTMS treatment was a more appropriate modification of GnP particles to improve the multifunctional properties of SR.

## 1. Introduction

In recent years, carbon nanofillers such as carbon nanofibers (CNFs) [1], carbon nanotubes (CNTs) [2] and graphene [3] have attracted significant interest all over the world, owing to their inherently high mechanical strength, and good electrical and thermal conductivity. Graphene, first discovered in 2004 [4] as a single atomic thick two-dimensional carbon layer, has extraordinary mechanical, thermal and electrical properties, and is considered to be a novel reinforcement in polymer nanocomposites [5]. Graphene nanoplatelets (GnPs) consist of multiple layers of graphene corresponding to partially exfoliated graphite [6]. Compared with single layer graphene, GnPs can be produced at large scale through top-down methods and commercially available with different particle sizes at a relatively low cost. As a result, GnPs are regarded as a promising carbon nanofiller for nanocomposites due to their good balance of properties and cost [7].

The overall reinforcement of graphene/GnP in polymer matrix depends greatly on the dispersion level of nanofillers and the interface interaction between two components [8,9]. However, graphene/GnP tends to form agglomerates through van der Waals force, which makes it difficult to disperse in the matrix [10]. Besides, graphene/GnP surface is rather atomically smooth and has a lack of active sites, probably resulting in a weak interfacial strength between the matrix and fillers [11]. The properties of the filled composite may be further enhanced if the fillers are properly modified. The approaches of modifying graphene/GnP can be mainly divided into two methods: (i) covalent bonding between the oxygen functional groups of graphene/GnP and modifiers; and (ii) non-covalent attachment of molecules on the surface of platelets. The former approach is confirmed to be an effective way to achieve the enhanced interfacial interactions between the filler and matrix, and further increases the mechanical and thermal performance [12,13]. Wang *et al.* [14] synthesized covalently functionalized graphene nanosheets (f-GNSs) by chemically grafting 3-aminopropyl triethoxysilane (APTS) and produced epoxy composite using f-GNSs as filler. It showed that the tensile strength of epoxy resins was increased by 45% at 1 wt % f-GNSs; meanwhile, the initial thermal degradation temperature of epoxy composite was improved by 20 °C at the same loading. Ma *et al.* [15] reinforced epoxy using GnP that covalently functionalized with a long-chain, end-amine surfactant, resulting in a significant increase in Young’s modulus and glass transition temperature of the modified GnP/epoxy composite. Although covalent functionalization of graphene/GnP can produce a good interface for graphene/GnP composites, the covalent bonding remains less than satisfactory due to the limited oxygen functional groups of graphene/GnP, or the unfortunate production of defect sites within the conjugated graphene sheet structure. As an alternative, non-covalent functionalization of graphene/GnP was carried out through physical absorption, including π-π stacking interactions and van der Waals force with surfactants or well-defined polymers. Wan *et al.* [16] treated graphene using a non-ionic surfactant to improve the compatibility and wettability with epoxy resin, and the strength of the composite filled with the treated graphene was greatly enhanced by 57% at 0.1 wt% compared to the neat epoxy. Teng *et al.* [17] used pyrene poly(glycidyl methacrylate) (Py-PGMA) to non-covalently functionalize GNSs; as a consequence, the thermal conductivity of Py-PGMA-GNS/epoxy was higher than that of the pristine GNS composite. However, to our knowledge, there are few reports on the study of the comparison of covalently and non-covalently modified graphene/GnP on the properties of silicone rubber (SR) composite.

Silicone rubber, one of the non-carbon skeleton polymers, has been widely used due to its excellent elasticity, physical and chemical properties, and good tolerance to extremely low and high temperatures [18]. Thus, based on these features, SR can be considered a good choice of elastomers in many extreme environments [19,20]. However, the applications of SR are usually limited by the low mechanical strength. Therefore, fillers (e.g. nanosilica, carbon black and clay) are usually incorporated into the SR matrix to improve its strength and stiffness. Compared with traditional fillers, graphene filled silicone composite might display multifunctional properties, *i.e.* increased thermal and electrical conductivity, increased thermal stability, and increased mechanical performance, which can meet the demanding requirements of new applications [21,22,23].

**Figure 1:**
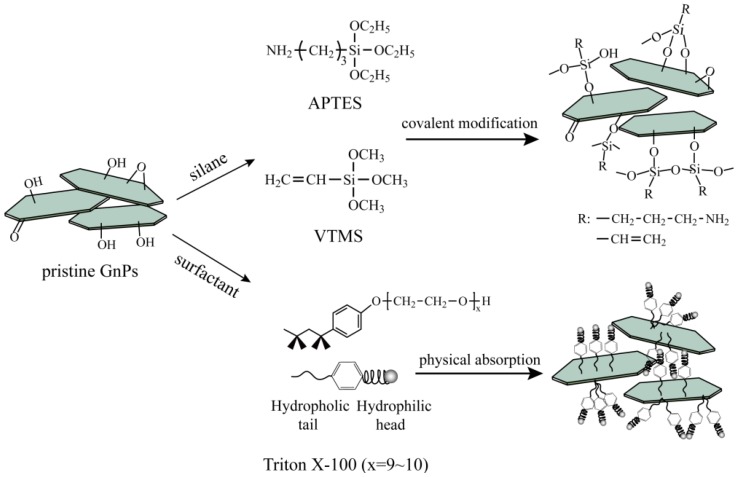
Schematic of the surface treatment of graphene nanoplatelets (GnP) particles with different modifiers.

In this work, we focus on the investigation of the effect of covalently and non-covalently functionalized GnP on the mechanical and thermal properties of SR composites. The covalent functionalization of GnP was conducted based on the reaction between hydroxyl groups on GnP and silane coupling agents. Moreover, the terminal moieties of silane coupling agents, chemical or non-chemical bonding, with the polymer matrix is also worth considering. Based on this idea, we chose common aminopropyltriethoxysilane (APTES) and vinyltrimethoxysilane (VTMS) as coupling agents. For non-covalent functionalization of GnP, nonionic surfactant Triton X-100 was selected to modify GnP through physical absorption since it has been proven to be effective in dispersing carbon materials [16,24]. The schematic of the surface treatment of graphene nanoplatelets is shown in Figure 1. The morphologies, and mechanical and thermal properties of the obtained SR composites were investigated to evaluate the effect of different treatments on the dispersion and interface in the composites.

## 2. Experimental

### 2.1. Materials

GnPs were provided by XG Sciences, Inc. (Lansing, MI, USA). They were ultra-thin particles having a platelet-like morphology with a lateral dimension of 5 μm and a thickness of 5–10 nm. Methyl vinyl silicone rubber (110-2; Mn = 500,000–700,000; vinyl content = 0.15–0.18 mol %) was purchased from BlueStar Chengrand Research Institute of Chemical Industry (Chengdu, China), and 2,5-bis(tert-butyl peroxy)-2,5-dimethyl hexane (DBPMH, Aladdin Industrial Inc., Shanghai, China) was used as vulcanizing agent. Aminopropyltriethoxysilane (APTES) and vinyltrimethoxysilane (VTMS) were kindly supplied by Heowns Biochemical Technology Co., Ltd. (Tianjin, China). Triton X-100 (polyoxyethylene octyl phenyl ether, POPE), methanol, ethanol, and tetrahydrofuran (THF) were all obtained from Sinopharm Chemical Reagent Co., Ltd. (Shanghai, China). Additionally, distilled water was used in the process of surface treated GnP. All reagents were used without further purification.

### 2.2. Preparation of GnP with Different Treatments

For the preparation of silane treated GnP, it was carried out in water/ethanol mixture. Firstly, 300 mg GnP was added into 300 mL ethanol-water under sonication for 30 min. Then, 450 mg APTES or VTMS was mixed with the above mixture at 65 °C, and the reaction was processed for 12 h under stirring. Subsequently, the mixture was filtered with filters with pore size of 0.22 μm. The products, aminopropyltriethoxysilane-GnP and vinyltrimethoxysilane-GnP, abbreviated as APTES-GnP and VTMS-GnP, respectively, were then washed several times with methanol and distilled water sequentially, and dried in a vacuum oven at 60 °C for 12 h.

For surfactant treated GnP, GnP (300 mg) was dispersed in 300 mL water containing 450 mg Triton X-100 and both sonicated for 30 min. The suspension was then kept at 65 °C for 12 h under stirring. GnPs possess a large surface area, and Triton X-100 could be physically absorbed on the platelets. The surfactant treated GnP, namely Triton-GnP, was obtained by filtration and washed with water, and then dried in a vacuum oven at 60 °C for 12 h.

### 2.3. Fabrication of GnP/SR Composites

The GnP/SR composites reinforced with 2 phr GnP were prepared by a solution blending method, which is described as following. SR was first dissolved in THF under continuous stirring to obtain a homogeneous SR solution. Meanwhile, GnPs were dispersed in THF (10 mg/mL) by sonication for 45 min. GnP/THF mixture was then added to the homogenous SR solution, and stirred for 30 min, followed by adding 1.5 phr DBPMH. The entire mixture was further sonicated for 2 h under stirring. THF was evaporated off by heating the mixture on a magnetic stir plate at 65 °C. Then, the high-viscosity mixture was dried in a vacuum oven at 60 °C for 24 h. The black gum was vulcanized in a mold at 165 °C and 10 MPa for 15 min. The secondary vulcanization process was carried out at 180 °C in a conventional oven for 2 h, and finally the GnP/SR composites were obtained. The neat SR was also prepared following the same procedure.

### 2.4. Characterization 

#### 2.4.1. Fourier Transform Infrared Spectroscopy (FTIR)

FTIR spectra were recorded from 500 to 4000 cm^−1^ at a resolution of 2 cm^−1^ using a Nicolet FTIR spectrophotometer (Nicolet 6700, Nicolet Instrument Company, Madison, WI, USA). The GnP particles were incorporated with KBr and pressed into a pellet for the measurement.

#### 2.4.2. Raman Spectroscopy

Raman spectroscopy characterization was obtained on a Renishaw inVia-Reflex (Gloucestershire, England, UK) with a 532 nm Ar laser. The Raman spectra of GnP were recorded in the range of 500–3500 cm^−1^ at room temperature.

#### 2.4.3. Morphology 

A field emission scanning electron microscopy (FESEM, Zeiss Ultra Plus, Carl Zeiss NTS GmbH, Oberkochen, Germany) was used to observe the morphology of pristine GnP operating at 5 kV. The tensile fracture surfaces of silicone-based samples were performed using a Quanta FEG450 environmental scanning electron microscope (ESME, FEI Company, Hillsboro, OR, USA) at an accelerated voltage of 15 kV. The samples of fracture surfaces were previously sputtered with a conductive layer of gold before imaging.

#### 2.4.4. Tensile Testing

The tensile properties were measured using a universal testing machine (Instron-4465, Instron Engineering Corporation, Norwood, MA, USA) with a crosshead speed of 500 mm/min according to GB/T 528-2009 [25] at room temperature. The dumbbell shape samples were prepared with dimension of 75 mm × 4 mm ×1.6 mm. Each group was tested at a minimum on three specimens.

#### 2.4.5. Thermogravimetric Analysis (TGA)

The thermal stability of the samples was performed on a TGA STA449c/3/G (NETZSCH Group, Selb, Germany). About 15 mg of sample was heated in an alumina crucible from ambient temperature to 800 °C in nitrogen atmosphere, with a heating rate of 10 °C/min.

#### 2.4.6. Differential Scanning Calorimetry (DSC)

DSC was applied to determine the crystallization and melting behavior of neat SR and its composites (Perkin Elmer Pyris1 DSC, Waltham, MA, USA). The sample was heated from −50 to 0 °C, and kept at 0 °C for 10 min to eliminate the thermal history. Then, the temperature was cooled down to −100 °C, followed by heating to 0 °C. All scans were conducted in nitrogen atmosphere and the heating/cooling rate was maintained at 10 °C/min.

#### 2.4.7. Thermal Conductivity 

Thermal conductivity of the composites was measured using a TC-7000H Laser flash apparatus (SINKU-RIKO, Chigasaki-shi, Japan) at room temperature. The samples were cut into square shape with length of 8 mm and thickness of 1.6 mm.

## 3. Results and Discussion

**Figure 2:**
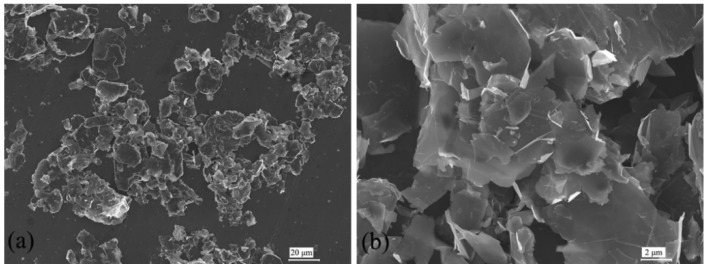
Scanning electron microscope (SEM) images of pristine GnP: (**a**) low magnification and (**b**) high magnification.

Figure 2 shows scanning electron microscope (SEM) micrographs of the pristine GnP particles. As can be seen in Figure 2a, GnP are small platelet-shape particles and have the tendency to form agglomerations due to their high surface area. In addition, note that the particle size ranges from 2 μm to 15 μm. The high magnification displayed in Figure 2b clearly shows the GnP agglomerates. Like other nano materials, it would be a great challenge to achieve uniform dispersion in polymers.

**Figure 3:**
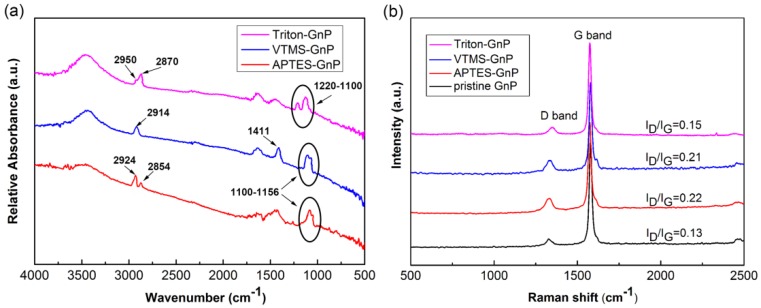
(**a**) Fourier transform infrared spectroscopy (FTIR) spectra of treated GnP after subtracting the reference spectrum of the pristine sample GnP; (**b**) Raman spectra of pristine and treated GnP.

FTIR and Raman spectroscopy were employed as measurements to monitor the functionalization of GnP. Figure 3a shows the FTIR spectra of treated GnP after subtracting the reference spectrum of the pristine sample GnP. As for the silane treated GnP, the peak at 1100–1056 cm^−1^ can be assigned to the symmetric and asymmetric stretching vibration of Si-O-Si, indicating the existence of silane hydrolysis products on the surface of GnP [14]. In the spectrum of APTES-GnP, the doublet located at 2924 and 2854 cm^−1^ corresponds to –CH_2_ asymmetric and symmetric stretching vibration, respectively [26]. However, the characteristic bands of –NH_2_ that should appear at around 3300 cm^−1^ was not observed, which is possibly attributed to overlapping with the peak of absorbed water [14]. In terms of VTMS treated GnP, the bands appearing at 1411 cm^−1^ and 2914 cm^−1^ can be assigned to vinyl groups and C–H vibration, respectively [27]. These findings probably suggest that chemical reactions between hydrolyzed silane and GnP took place. Unlike silane treatment, Triton X-100 surfactant was physically absorbed on the surface of GnP through hydrophobic segments, the peaks at 1220–1100 cm^−1^ can be assigned to C–O stretching bands, reflecting Triton X-100 molecules attached on the surface of GnP [28]. The absorption peak at 2870 cm^−1^ and a small shoulder at 2950cm^−1^ probably correspond to the symmetric and asymmetric stretching vibration of –CH_3_ and –CH_2_ groups from Triton X-100, respectively; nevertheless, it is difficult to distinguish the stretching vibration of –CH_3_ with –CH_2_ groups because they are very close to each other [16,28]. The Raman spectra of pristine and treated GnP are shown in Figure 3b. The obvious absorptions at 1353 cm^−1^ and 1576 cm^−1^ correspond to the D band and G band, respectively. D band refers to disorder of the in-plane graphene structure and G band relates to the sp^2^ resonance on an ordered graphitic lattice. The intensity ratio of D band to G band (*I*_D_/*I*_G_) generally represents the density of defects [15]. The surfactant treatment slightly increases the *I*_D_/*I*_G_, indicating no significant change in the structural integrity of GnP after being absorbed with Triton X-100. Through silanization, the *I*_D_/*I*_G_ increases from 0.13 to 0.22, which reveals that the covalent functionalization can introduce a relatively high level of defects in GnP.

**Figure 4:**
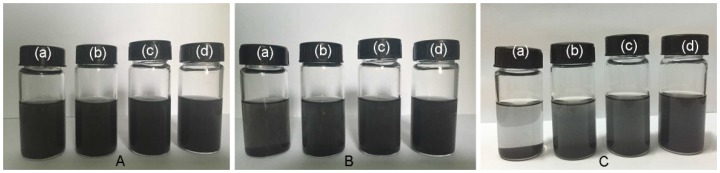
Tetrahydrofuran (THF) dispersion of pristine GnP (**a**); aminopropyltriethoxysilane-GnP (APTES-GnP) (**b**); vinyltrimethoxysilane-GnP (VTMS-GnP) (**c**); and Triton-GnP (**d**) after standing for different times: (**A**) 0 h; (**B**) 1 h; and (**C**) 12 h.

Figure 4 shows the dispersion state of the pristine GnP, APTES-GnP, VTMS-GnP and Triton-GnP in THF. Apparently, the pristine GnP precipitated in THF only after standing for 1 h, while the treated GnP showed very little sediment. After standing for 12 h, the pristine GnP deposited completely and formed a transparent liquid on the top. The silane modified GnP largely settled on the bottom and the upper portion became partially visible grey, while the Triton-GnP dispersion remained stable in the environment. This confirms that the modified GnP shows better dispersion stability than the pristine GnP, and is more evidence for the superiority of Triton treated GnP.

**Figure 5:**
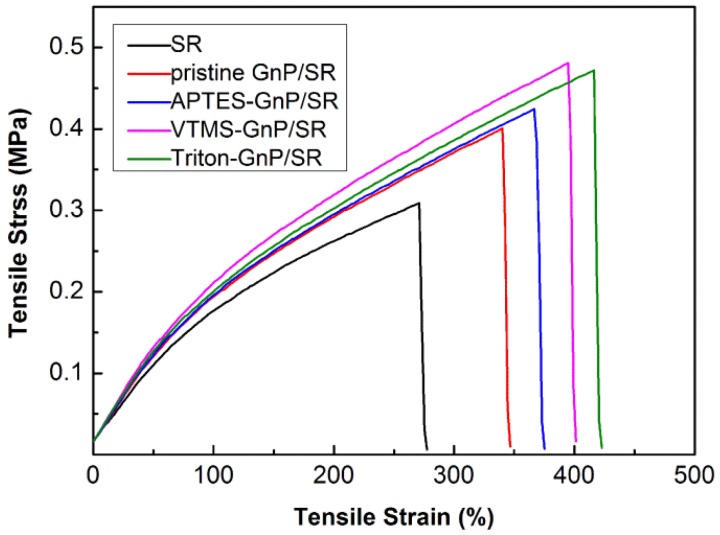
Typical stress-strain curves of neat silicone rubber (SR) and the composites filled with various GnP.

**Figure 6:**
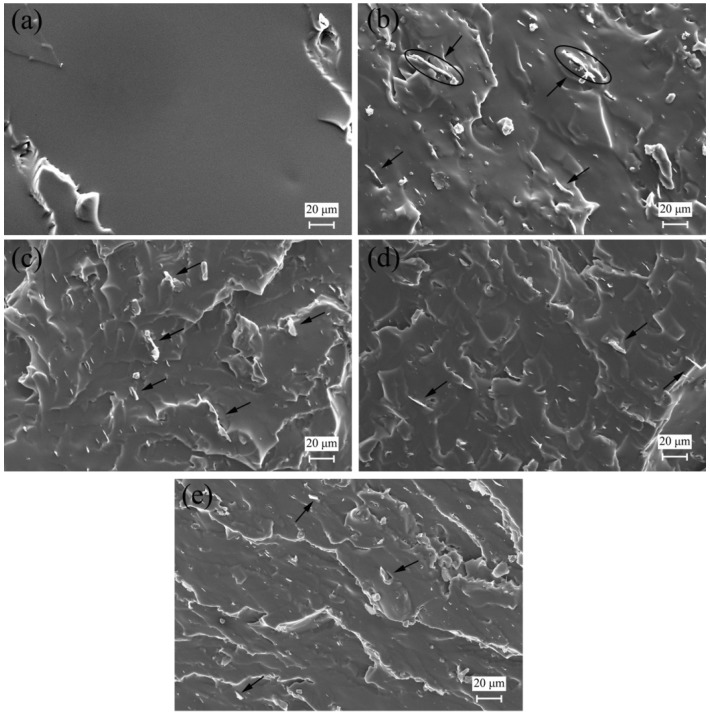
Field emission scanning electron microscopy (ESEM) images of the tensile fracture surfaces of neat SR and the composites: (**a**) SR; (**b**) pristine GnP/SR; (**c**) APTES-GnP/SR; (**d**) VTMS-GnP/SR; and (**e**) Triton-GnP/SR composites.

Figure 5 depicts the typical stress-strain curves of the neat SR and its composites. The detailed data of tensile strength, elongation at break and tensile modulus (stress at 100 and 300% strains) of the neat SR and its composites are summarized in Table 1. The addition of GnP improves the tensile strength as well modulus, and the reinforcement is more pronounced in the modified GnP composites [9]. As presented in Figure 5 and Table 1, the tensile strength of the neat SR and its composites follows the order: SR < pristine GnP/SR < APTES-GnP/SR < Triton-GnP/SR < VTMS-GnP/SR. Compared with the neat SR, the tensile strength of the pristine GnP/SR composite is increased by 29% from 0.31 to 0.40 MPa, whereas that of the composite with VTMS-GnP shows an improvement of 58%. The platelets can form physical entanglement with the silicone chains, and transfer the load from the matrix to the platelets; nevertheless, the poor dispersion of GnP and weak interfacial adhesion limit the reinforcement of pristine GnP. The Si–CH_2_=CH_2_ groups anchored on VTMS-GnP surface were expected to form some firm chemical links with the silicone chains, resulting in a good interfacial interaction between the platelets and matrix, which was more efficient at transfering the stress from the silicone to the platelets interface, and therefore increased the tensile strength the most. However, chemical bonding between the APTES treated GnPs and the SR cannot be formed, and the number of active sites on the pristine GnP surface is limited, so the improvement of this silane treatment is not obvious. To our surprise, the reinforcement of surfactant treatment on the mechanical performance is almost the same with the VTMS coupling agent. The hydrophobic octyl group of the surfactant can be absorbed on the surface of the platelets, while the hydrophilic segment interacts with silicone through hydrogen bonding [29]. The surfactant acts as a bridge between the GnPs and rubber matrix, resulting in improved compatibility and wettability of the platelets, which provides a good adhesion to the silicone (see Figure 6e). The elongation at break of the GnP/SR composites is remarkably increased, possibly arising from the slippage of the rubber chains along the platelets during the tensile stretching; the value of the VTMS-GnP and Triton-GnP composites were increase by 133% and 148%, respectively, when compared to the neat SR, much higher than that of the pristine GnP composite. The more prominent reinforcement of VTMS-GnP and Triton-GnP is attributed to the good dispersion of GnP and enhanced interfacial interactions between GnP and silicone matrix.

**Table 1:** Tensile properties of neat SR and the composites filled with various GnP.

Sample	Tensile Strength (MPa)	Elongation at Break (%)	100% Modulus (MPa)	300% Modulus (MPa)
SR	0.31 ± 0.028	273 ± 9	0.175	-
Pristine GnP/SR	0.40 ± 0.031	344 ± 12	0.193	0.367
APTES-GnP/SR	0.43 ± 0.025	372 ± 7	0.195	0.375
VTMS-GnP/SR	0.49 ± 0.019	406 ± 5	0.212	0.405
Triton-GnP/SR	0.48 ± 0.015	421 ± 11	0.199	0.387

The morphologies of the tensile fracture surfaces of the neat SR and its composites are displayed in Figure 6. The fracture surface of the neat SR is quite smooth and flat, while the composites show more rough and tortuous pathways. Since the pristine GnP is not compatible with the silicone matrix, some platelets stack to produce high thickness (see the circle in Figure 6b). In addition, the fracture surfaces of the composites containing pristine GnP and APTES-GnP show more platelets projecting outside from the surfaces (see the arrow in Figure 6), meaning a weak adhesion to the matrix. In contrast, the VTMS-GnP and Triton-GnP seem to be well embedded in the silicone matrix, an indication of better adhesion to the rubber. All these observations confirm the good interfacial interactions between the SR and VTMS-GnP or Triton-GnP are in good agreement with the high mechanical properties.

**Figure 7:**
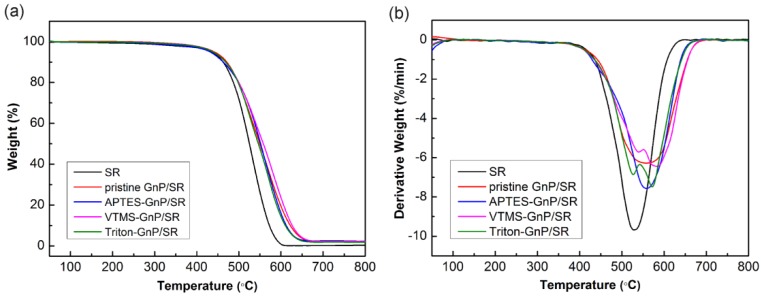
Thermogravimetric analysis (TGA) and differential thermogravimetric analysis (DTGA) curves of neat SR and the composites with different GnP measured in nitrogen atmosphere: (**a**) TGA curves and (**b**) DTGA curves.

Figure 7 shows the TGA curves and corresponding first differential thermogravimetric analysis (DTGA) curves of the neat SR and the composites. The initial decomposition temperature (*T*_0.1_) defined as 10% weight loss temperature, the temperature of 50% weight loss (T_0.5_) as the mid-point of decomposition, and the temperature of maximum decomposition rate (T_max_) are listed in Table 2. The value in the bracket means the increase of temperature compared with the corresponding temperature of SR. It can be seen that the T_0.1_ of the neat SR appears at 462.5 °C, and then a rapid weight loss centers at 528.2 °C, corresponding to the decomposition of the siloxane chains. For filled samples, the *T*_0.5_ and *T*_max_ shifts towards higher temperatures, indicating that the addition of GnP can improve the thermal stability significantly. The *T*_0.5_ and *T*_max_ of the pristine GnP composite are 549.1 and 568.3 °C, respectively; an increase of 23.7 and 40.1 °C, respectively, compared with the neat SR. The incorporation of two-dimensional GnP is postulated to form a platelet barrier effect, slowing the escape of volatile decomposition products, and thereby increasing the thermal stability. Similar results were reported in a polymer/nanoclay composite [30]. Besides, GnP with high thermal conductivity might act as a heat dissipater and help in transferring and diffusing the thermal energy, which reduces the heat concentration on the matrix, thus moving the T_max_ to higher temperatures [31]. This effect in improved thermal stability was also confirmed in the CNT filled polymer system [32]. As seen in Figure 7 and Table 2, the VTMS treated GnP composite has improved thermal stability, while that of the APTES and Triton treated GnP composites are almost unchanged compared to the pristine GnP composite. This may be due to the chemical links between VTMS-GnP and silicone leading to an increase in the rigidity of siloxane chains, and also due to the good VTMS-GnP/silicone interaction that offers a better barrier effect. Moreover, the thermal degradation of the composite with pristine GnP or APTES-GnP shows a one-step process like neat SR, suggesting that the incorporation of the two type GnP particles does not alter the degradation nature of silicone. In the case of the VTMS-GnP and Triton-GnP composites, a small shoulder below 550 °C in the DTGA curves appeared, implying that a good interface interaction induces the difference in degradation behavior, which is consistent with the ESEM observations.

**Table 2:** Characteristic TGA temperature data of neat SR and the composites with different GnP measured in nitrogen atmosphere.

Sample	*T*_0.1_ (°C)	*T*_0.5_ (°C)	*T*_max_ (°C)
SR	462.5	525.4	528.2
Pristine GnP/SR	471.5 (+8.9)	549.1 (+23.7)	555.3 (+30.1)
APTES-GnP/SR	462.6 (+0.1)	551.4 (+26.0)	558.4 (+30.2)
VTMS-GnP/SR	468.7 (+6.2)	558.7 (+33.3)	583.6 (+55.4)
Triton-GnP/SR	468.6 (+6.1)	546.2 (+20.8)	572.1 (+43.9)

**Figure 8:**
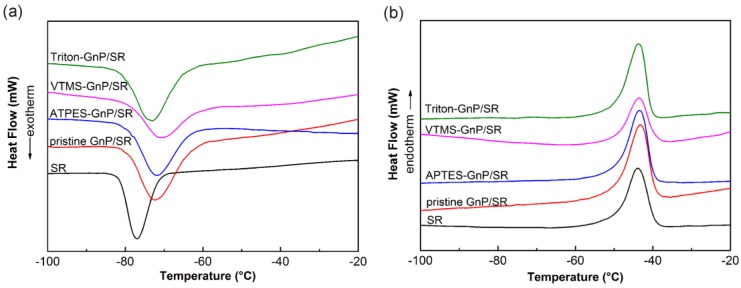
Crystallization (**a**) and melting (**b**) curves of neat SR and the composites with different GnP.

The influence of GnP (pristine or treated) on the crystallization and melting behavior of SR was investigated with DSC analysis, and the results are shown in Figure 8 and Table 3. In the DSC crystallization curve (Figure 8a), an exothermic peak of the neat SR around at −76.88 °C is observed, reflecting the crystallization of silicone. The crystallization temperatures (*T*_c_) of the filled silicone shift to higher temperatures, indicating that GnP here could facilitate faster crystallization of the molten chains and GnP substantively acts as a nucleating agent [33,34]. The *T*_c_ of the APTES or Triton treated composite is comparable to the value of the pristine GnP/SR composite. However, the VTMS-GnP/SR composite shows a much higher value, and it could be inferred that heterogeneous nucleation of VTMS-GnP/SR composite is much faster, which is ascribed to the chemical links between two components. The crystallization degree of all composites, as determined from the heat of fusion (Δ*H*_m_), shows a reduction compared to the neat SR. The addition of laminar GnP may impede the rearrangement of some silicone chains during the crystallization process. Menes *et al.* also reported the decreased crystallinity in the thermoplastic polyurethane/ultra-thin graphite nanocomposites [35]. The decrease of crystallinity is more significant in the composite with VTMS-GnP; that is, the hindrance effect of GnP covalently linked to silicone is much stronger. These results imply that the chemical links between GnPs and silicone play an important role on the behavior of crystallization. According to Table 3, the melting temperatures of the GnP/SR composites do not change considerably compared to the neat SR, which indicates that GnP does not correlate with the crystal structure.

**Figure 9:**
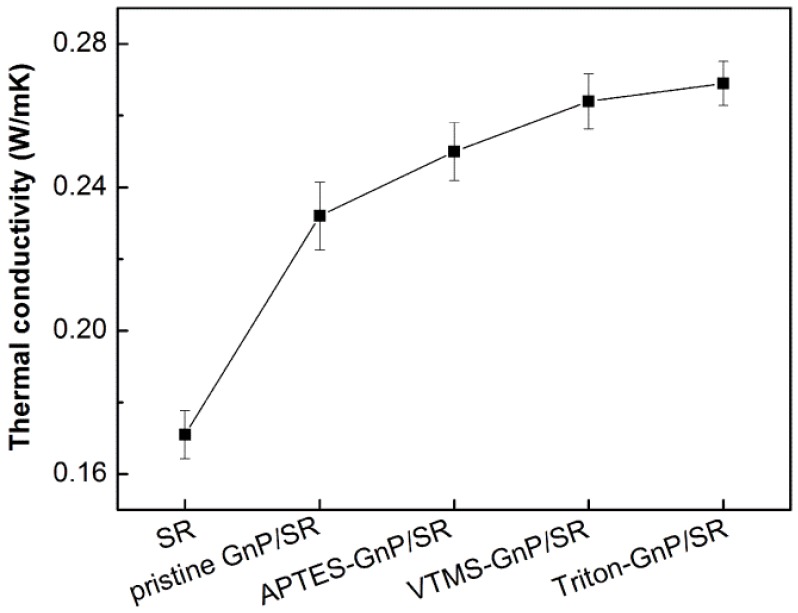
Thermal conductivity of neat SR, pristine GnP, APTES-GnP, VTMS-GnP and Triton-GnP composites.

It is known that the thermal conductivity depends on the content, dispersion of fillers, and the thermal contact resistance of interface between the fillers and matrix [36,37,38]. For amorphous polymers, the phonons play an important role in the heat conduction [3]. In order to improve the thermal conductivity of the composites, a decrease in the phonon scattering or acoustic impedance mismatch at the interface between the matrix and fillers is needed, and as a result the functionalization of fillers seems to be a feasible approach. For example, Ganguli *et al.* [39] treated exfoliated graphite using 3-aminopropoxyltriethoxy silane, and the thermal conductivity of the treated composite was almost double that of the untreated composite at 8% filler loading. Likewise, the thermal conductivity of silicone composite would be further increased after the treatment of GnP particles. The effect of unmodified and modified GnP on the thermal conductivity of silicone composite was also studied and the results are presented in Figure 9. The thermal conductivity of the neat SR is 0.171 W/mK; in the presence of pristine GnP, the value arises to 0.232 W/mK, an improvement of 35.7%. For modified GnPs, the thermal conductivity of APTES-GnP/SR, VTMS-GnP/SR, and Triton-GnP/SR composites increases 46.2%, 54.4% and 57.3%, respectively, as compared to the neat SR, which is relatively higher than that of the pristine GnP/SR composite. The thermal conductivity of VTMS-GnP/SR composite is superior to the APTES treated counterpart, mainly due to a better interfacial adhesion between SR and VTMS-GnP resulting from the formation of chemical links between VTMS-GnP surface and silicone chains. The enhanced interfacial adhesion reduces phonon scattering, leading to higher thermal conductivity. Interestingly, the composite filled with surfactant treated GnP shows marginally better performance in thermal conduction than the VTMS treated GnP composite. As mentioned before, the Triton treated GnP had a little higher structural integrity than the silane treated GnP, which was beneficial for increasing heat transfer. This result further confirms that the surfactant Triton X-100 treated GnP has fine dispersion and interfacial quality in the SR composite.

**Table 3:** Values for crystallization temperature (*T*_c_), melting temperature (*T*_m_) and heat of fusion (Δ*H*_m_) for neat SR and its composites.

Sample	*T*_c_ (°C)	*T*_m_ (°C)	Δ*H*_m_ (J/g)
SR	−76.88	−44.41	25.37
Pristine GnP/SR	−72.36	−43.31	23.34
APTES-GnP/SR	−71.92	−43.44	22.97
VTMS-GnP/SR	−70.92	−43.56	20.49
Triton-GnP/SR	−72.93	−43.64	22.26

## 4. Conclusions

In this work, the effects of GnP with different treatments on the mechanical and thermal properties of GnP/SR composites were investigated. It has been found that the SR composite showed a considerable improvement in the mechanical properties and thermal conductivity after surface modification of GnP. For the silane treatment, the increase in mechanical properties and thermal conductivity of the VTMS-GnP composite were much higher than that of the APTES treated counterpart, which was due to the stronger interfacial interactions between VTMS-GnP and silicone chain resulting from the formation of chemical bonds. The Triton X-100 modified GnP composite exhibited comparable mechanical properties compared to the VTMS-GnP/SR composite. It is also noted that the VTMS treated GnP composite demonstrated the highest thermal stability and crystallization temperature among the four types of composites. This study suggested that GnP modified with VTMS was more effective in improving the multifunctional properties of SR. The surfactant Triton X-100 treatment also provided us with a potential way to fabricate GnP composites with improved properties.
